# Supported Ir-based anode catalysts for proton exchange membrane water electrolysis: recent progress and perspectives

**DOI:** 10.3389/fchem.2026.1874443

**Published:** 2026-08-07

**Authors:** Huawei Huang, Youping Li, Bo Xu, Yun Tan, Sumin Guan, Lu Song

**Affiliations:** 1 China Yangtze Power Co., Ltd., Wuhan, China; 2 Department of Materials Science and Engineering, Korea Advanced Institute of Science and Technology (KAIST), Daejeon, Republic of Korea

**Keywords:** hydrogen production, low-iridium anodes, oxide supports, proton exchange membrane water electrolysis, supported Ir catalysts

## Abstract

Proton exchange membrane water electrolysis (PEMWE) is a promising green technology for hydrogen production. It enables operation at high current densities, rapid dynamic response, and the generation of high-purity hydrogen. However, the large-scale commercialization of this technology is constrained by the scarcity and high cost of iridium (Ir) which is a rare metal that serves as the most reliable anode catalyst for the acidic oxygen evolution reaction (OER). Consequently, supported iridium-based catalysts have been proposed as a crucial strategy to reduce iridium loading while simultaneously maintaining catalytic activity and durability. Dispersing iridium species onto suitable support materials not only enhances iridium utilization efficiency but also effectively modulates the local coordination environment, electronic structure, and interfacial stability of the active sites. This mini-review compares TiO_2_-, SnO_2_-, and other oxide-supported Ir catalysts through four linked questions: how the support controls Ir dispersion and working-state structure; how conductivity and corrosion resistance constrain catalyst-layer construction; how support chemistry affects activity and dissolution; and whether half-cell gains are retained in low-Ir membrane electrode assemblies. The resulting framework highlights the dispersion-stability trade-off, support degradation, and incomplete translation from model electrodes to practical devices.

## 1 Introduction

**FIGURE 1:**
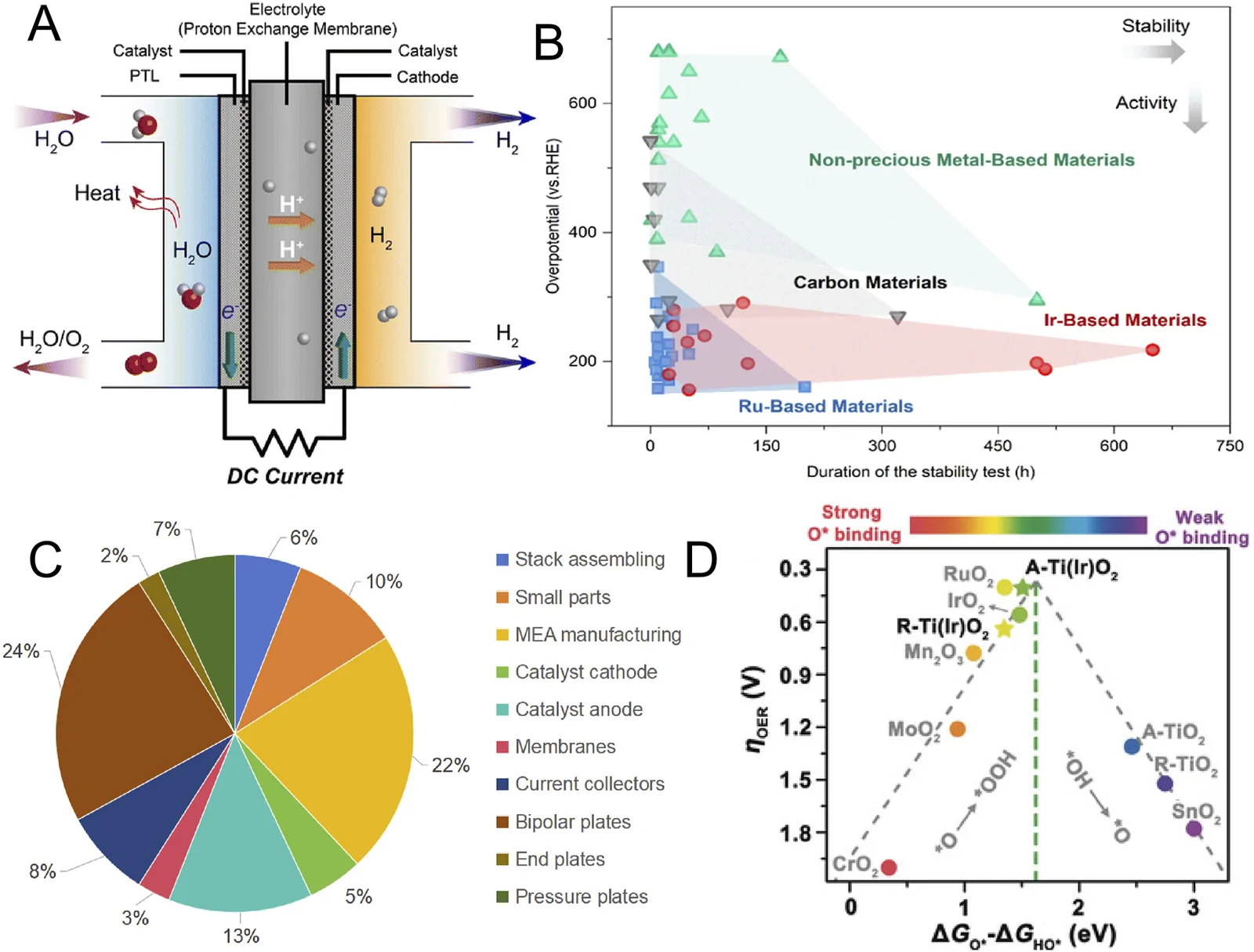
**(A)** Schematic diagram of a PEM electrolyzer. Reproduced with permission from (Song et al., 2025). **(B)** Stability test results and overpotentials at 10 mA cm^-2^ of recently reported catalysts. Reproduced from (Ma and Mu, 2023), licensed under CC BY 4.0. **(C)** Cost proportion analysis of components after the mass production of PEM electrolyzers. **(D)** A volcano plot depicting the theoretical overpotentials of the OER on various materials as a function of their △*G*_O*_-△*G*_HO*_ values. Reproduced with permission from (Chen et al., 2020).

Proton exchange membrane water electrolysis (PEMWE) is widely regarded as one of the most promising green hydrogen production technologies. It operates at high current densities, yields high-purity hydrogen, and responds rapidly to fluctuating renewable electricity supplies (Johnson et al., 2025; Shi et al., 2024). However, the oxygen evolution reaction (OER) occurring at the anode side occurs under strongly acidic and oxidative conditions (Figure 1A), which places high demands on catalyst activity and, more importantly, poses a major challenge to long-term stability (Song et al., 2025; Geuß et al., 2024). Among the various acidic OER catalysts, iridium (Ir)-based materials remain the most reliable choice, striking the optimal balance between activity and durability (Figure 1B). (Ma and Mu, 2023; Salemi et al., 2024; Cao et al., 2026; Zhao et al., 2026; Xiong et al., 2024) However, the large-scale commercialization of PEMWE technology is fundamentally constrained by the scarcity and high cost of Ir resources. At current Ir based catalyst loadings, this amount of Ir can support an annual production capacity of only about 6.1 GW, far below the 2030 target of 100 GW (Zhang et al., 2026). In addition, techno-economic analysis shows that under large-scale manufacturing conditions, the cost of the anode catalyst can account for about 13% of the total PEM electrolyzer stack cost (Figure 1C). (Böhm et al., 2019; Huang et al., 2024) Therefore, reducing Ir loading while maintaining high activity and high durability has become a central goal in PEMWE anode design. In this context, supported Ir based catalysts have emerged as a promising strategy and a key route to achieving low Ir loading PEMWE anodes. By dispersing Ir species as nanoparticles, sub nanometer clusters, or isolated atoms on suitable supports, these systems can maximize the proportion of electrochemically accessible Ir sites, thereby reducing the total amount of precious metal used. More importantly, in PEMWE systems, the support not only provides physical anchoring. It also significantly influences the dispersion state, local coordination environment, electronic structure, and redox behavior of the Ir species, thus simultaneously tuning both catalyst activity and durability (Figure 1D). (Chen et al., 2020; Yuan et al., 2023) Yet support selection cannot be reduced to choosing the most conductive or corrosion-resistant oxide. A viable support must retain Ir under oxidative polarization, sustain electronic percolation at low loading, and remain compatible with ionomer and porous transport pathways (Feng et al., 2025; Spöri et al., 2017; Wang et al., 2025). Existing reviews generally address Ir-based acidic OER catalysts, PEMWE components, or catalyst-layer engineering at broader scope (Song et al., 2025). Here, we focus specifically on oxide-supported Ir anodes and organize the literature from support chemistry, through working-state Ir structure, to low-Ir MEA performance. This framework directly compares TiO_2_, SnO_2_/ATO, and emerging oxides while identifying where activity gains are offset by support corrosion, Ir restructuring, or electrode-level transport losses.

## 2 Oxide-supported Ir catalysts for PEMWE

Supported architectures reduce precious-metal demand only when dispersion, interfacial stabilization, and electrode connectivity improve together. Nanoparticles, subnanoclusters, and isolated atoms increase accessible Ir, but the support determines whether these sites remain connected and stable during acidic OER (Li et al., 2024; Ni et al., 2025; Shi et al., 2025). The largest mass-activity gains often occur at low loading, where catalyst-layer resistance and local degradation become most consequential (Carmo et al., 2013; Tran et al., 2024). These supports therefore cannot be ranked by one descriptor. TiO_2_ offers acid resistance and compatibility with IrO_x_ but poor conductivity; SnO_2_/ATO improves charge transport but may lose Sn or dopants; ZrO_2_, CeO_2_, and mixed oxides regulate oxygen or Ir valence more directly, although scalable electrode integration is less established (Chen et al., 2025; Fang et al., 2024; Gong et al., 2025; Moriau et al., 2022; Park et al., 2025; Zaman et al., 2024). We compare them by Ir utilization, control of the working state, support stability, and device-level performance.

### 2.1 Titanium oxide supported Ir catalysts

Titanium oxide is among the most extensively investigated supports for PEMWE anodes because it combines exceptional corrosion resistance in acidic environments with strong structural compatibility with IrO_x_. Rutile TiO_2_ is particularly attractive owing to its crystallographic match with rutile IrO_2_, which promotes close interfacial contact and helps stabilize highly dispersed Ir species (Tran et al., 2024). This feature makes TiO2 fundamentally different from carbon-based supports, which, although more conductive, generally do not remain sufficiently stable under anodic OER conditions. Recent studies have further shown that TiO_2_ can accommodate several useful structural configurations for low-Ir catalysts (Bernt and Gasteiger, 2016; Pak et al., 2025; Pham et al., 2020). One representative approach is the TiO_2_@IrO_x_ core-shell configuration, in which TiO_2_ particles are coated with a thin IrO_x_ layer rather than decorated with large Ir particles (Hoffmeister et al., 2024; Pham et al., 2020; Tran et al., 2024). Hoffmeister et al. synthesized such a TiO_2_@IrO_x_ core-shell catalyst by photodeposition and obtained a uniform ultrathin IrO_x_ shell of about 2.1 nm (Hoffmeister et al., 2024). This structure markedly improved Ir utilization while maintaining favorable catalytic stability.

**FIGURE 2:**
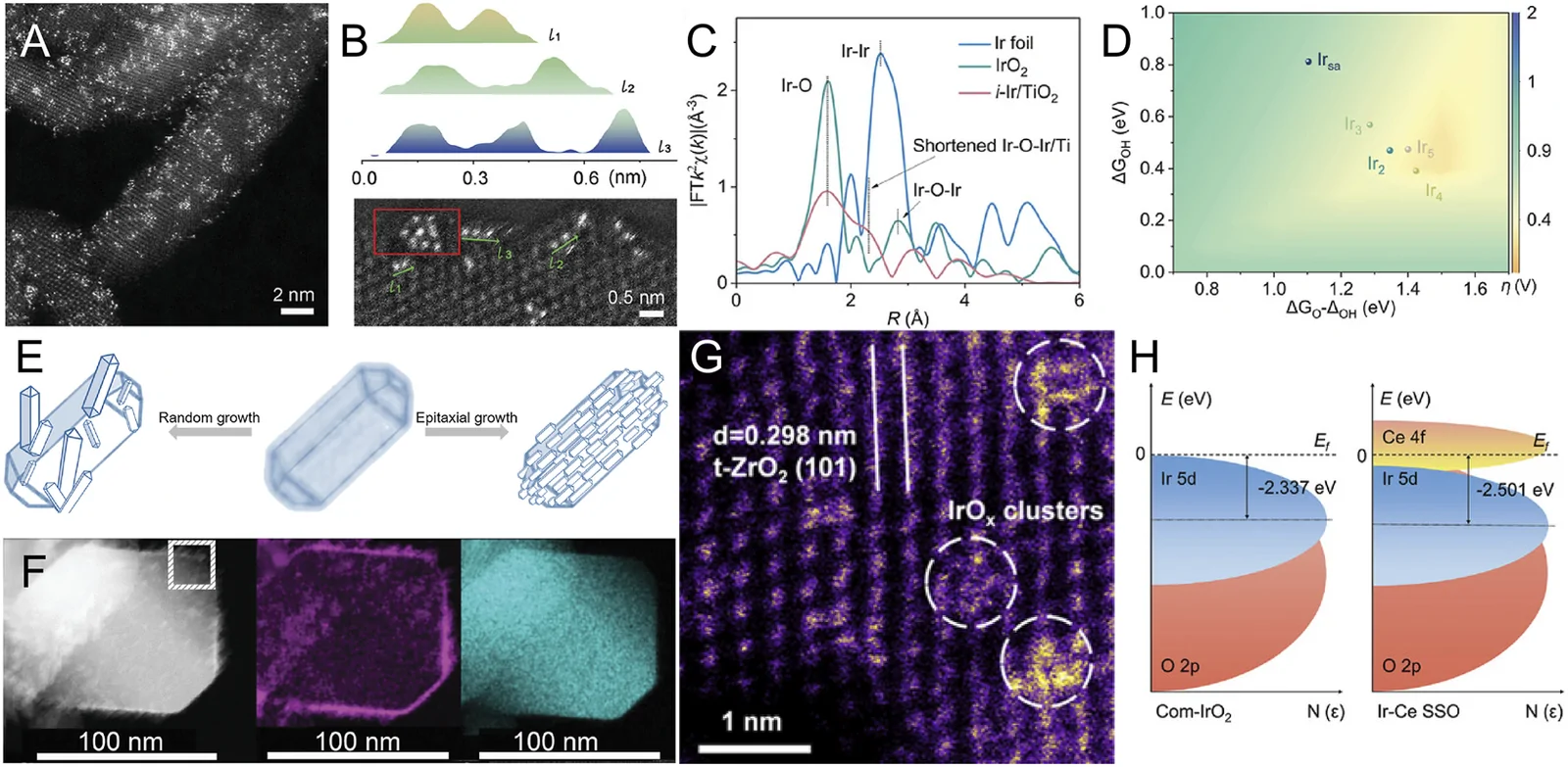
**(A, B)** HAADF-STEM images and the corresponding pseudocolor surface images of i-Ir/TiO_2_. **(C)** FT-EXAFS curves at the Ir L_3_-edge of the Ir foil, IrO_2_, and i-Ir/TiO_2_. **(D)** 2D map of theoretical overpotentials as a function of △*G*_O*_–△*G*_OH*_ and △*G*_OH*_. Reproduced with permission from (Li et al., 2025). **(E)** Simplified crystallization pathways of amorphous IrO(OH)_x_ on crystalline MO substrates: random IrO_2_ growth (left) vs. epitaxial growth along the substrate (right). **(F)** HAADF-STEM images and the corresponding EDX elemental mapping of IrO_2_@SnO_2_. Reproduced from (Kost et al., 2024), licensed under CC BY 4.0. **(G)** HAADF-STEM image of IrO_x_/t-ZrO_2_. Reproduced with permission from (Fang et al., 2025). **(H)** Schematic representation of the band structure for Ir-Ce substitution solid solution oxide (SSO) and Com-IrO_2_. Reproduced with permission from (Dong et al., 2024). Taken together, A-H figures illustrate three support functions: control of Ir dispersion and coordination, crystallographic anchoring and connectivity, and regulation of oxygen or electron transfer.

Beyond geometric dispersion, an increasingly important strategy is to use TiO_2_ as an active interfacial platform that can anchor and electronically regulate Ir species (Böhm et al., 2021; Kidtang et al., 2025; Li et al., 2025; Shin et al., 2025). For example, Li et al. reported a TiO_2_-supported catalyst containing spatially correlated Ir atoms embedded within the TiO_2_ lattice (i-Ir/TiO_2_) (Figures 2A,B). Structural analysis revealed distinct Ir–O coordination and shortened Ir–O–Ir(Ti) environments (Figure 2C), indicating strong local coupling across the Ir-support interface (Li et al., 2025). The resulting flexible Ir–O–Ir configuration enhanced intrinsic OER activity by tuning the *d*-band center of Ir sites and optimizing the adsorption of reaction intermediates (Figure 2D). In addition, modified TiO_2_ supports with improved electronic properties have attracted increasing attention. For example, Zhu et al. found that Nb-doped TiO_2_ could stabilize Ir nanoparticles through strengthened catalyst-support interaction (Zhu et al., 2025). These studies indicate that the role of TiO_2_ extends beyond chemical inertness; more importantly, it can serve as a tunable electronic scaffold for interfacial Ir stabilization and activity regulation.

These advantages do not remove the main device-level limitation of TiO_2_: a catalyst active in a thin model film may become partly inaccessible in a low-Ir MEA. Because TiO_2_ contributes little conductivity, incomplete through-plane percolation can increase ohmic loss and leave isolated Ir domains underused; additional ionomer may improve proton access but block pores and hinder oxygen removal (Bernt and Gasteiger, 2016; Pak et al., 2025; Pham et al., 2020). Practical designs therefore need ultrathin shells or interconnected IrO_x_ pathways, stable conductive dopants, and controlled support packing. The target is not simply the smallest Ir particle size, but a catalyst layer that balances Ir accessibility, proton conduction, gas transport, and mechanical integrity.

### 2.2 Tin oxide-supported Ir catalysts

The conductivity limitation of TiO_2_ explains the interest in tin oxide-based supports, particularly antimony-doped tin oxide (ATO). Doping gives SnO_2_ higher electronic conductivity and can improve utilization of dispersed Ir in low-loading electrodes (Cai et al., 2025; Jiménez-Morales et al., 2025; Kost et al., 2024). This benefit is coupled to a less favorable stability profile: Sn and dopant species may dissolve, redistribute, or alter surface termination under anodic polarization (Zaman et al., 2024; Moriau et al., 2022; da Silva et al., 2020). Tin oxide therefore provides a useful counterpoint to TiO_2_, offering stronger charge transport but requiring conductivity to be retained without creating a support-driven degradation pathway.

The seminal work by Oh et al. established the technological importance of this support family. In that study, Ir nanodendrites supported on ATO significantly outperformed commercial Ir catalysts, reaching 1.5 A cm^-2^ at 1.80 V in a PEM electrolyzer while maintaining excellent durability under constant-current operation (Oh et al., 2015). This work was important not only because of its high activity, but also because it demonstrated that oxide-supported Ir catalysts could translate half-cell advantages into device-level PEMWE performance. Subsequent studies further reinforced the case for ATO. For example, Hartig-Weiss et al. reported a high-surface-area, highly conductive ATO support enabled an Ir/ATO catalyst with an OER activity of about 1100 A g_Ir_^−1^ at 1.45 V_RHE_, which was attributed to high Ir dispersion and strong catalyst-support interaction (Hartig-Weiss et al., 2020). Follow-up mechanistic studies further suggested that the exceptional activity and stability of Ir/ATO are closely related to support-regulated Ir dispersion, oxidation state, and interfacial coupling (Saveleva et al., 2020). Taken together, the device-level current density and half-cell mass activity demonstrate the benefit of conductive Ir dispersion, but they are not interchangeable: the former includes catalyst-layer transport, whereas the latter mainly reflects accessible Ir under model conditions (Hartig-Weiss et al., 2020; Oh et al., 2015; Saveleva et al., 2020).

More recently, the role of tin oxide has expanded beyond that of a conductive scaffold. Kost et al. showed that, because SnO_2_ and IrO_2_ share the rutile structure with only a small lattice mismatch, SnO_2_ can direct the chemically epitaxial growth of crystalline IrO_2_, producing interconnected and firmly anchored IrO_2_ nanocrystals on the support surface (Figures 2E,F). (Kost et al., 2024) This crystallographically matched architecture improved conductivity and stability at high overpotentials even at very low Ir contents, highlighting crystal-structure compatibility as an additional design lever in tin oxide-supported systems.

At the same time, this support family clearly illustrates the central trade-off in supported PEMWE catalysts: the same catalyst-support interaction that enhances activity may also introduce new degradation pathways. Later studies showed that the bulk and surface structures of ATO strongly influence supported Ir performance, indicating that support crystallinity, dopant distribution, and surface termination are decisive design variables rather than secondary details. More importantly, some reports suggested that catalyst-support interaction can also contribute to the deactivation of IrO_x_/Sb:SnO_2_ catalysts, while others observed higher Sn dissolution in supported systems than in unsupported IrO_2_ under certain conditions (Jang et al., 2022). These results make clear that ATO is not inherently stable simply because it is a conductive oxide; rather, its performance depends on whether conductivity, dopant chemistry, and anodic stability can be balanced simultaneously.

Overall, tin oxide-supported Ir catalysts remain highly valuable for low-Ir PEMWE anodes, but the design focus has shifted over time. Early studies mainly emphasized maximizing conductivity and Ir dispersion, whereas current efforts increasingly prioritize support stabilization and interfacial regulation through dopant optimization, synthetic control, and crystallographically guided IrO_2_ growth. From a practical perspective, tin oxide-based supports have already provided strong proof-of-concept for supported low-Ir PEMWE anodes, but their long-term commercial relevance will ultimately depend on whether support corrosion can be controlled as effectively as Ir dissolution.

### 2.3 Other oxide-supported Ir catalysts

Where TiO_2_ prioritizes corrosion resistance and SnO_2_/ATO prioritizes conductivity, emerging oxides seek more direct control of Ir valence, oxygen transfer, and lattice confinement. These include zirconium oxide, cerium oxide, manganese oxide, Ta-containing oxides, and mixed-oxide hosts with specific lattice or interfacial interactions (Dong et al., 2024; Fang et al., 2025; Hao et al., 2021; Li et al., 2024; Paulo et al., 2024; Shen et al., 2026; Wu et al., 2024). Although this category is more diverse and less mature than the titanium oxide and tin oxide families, it remains scientifically important because it has provided some of the clearest evidence that support chemistry can fundamentally reshape the electronic structure and durability of Ir, rather than merely improve its dispersion.

One emerging direction is the use of nontraditional oxide hosts that can lattice-anchor isolated or highly dispersed Ir species under acidic OER conditions. A representative example is tetragonal zirconia (t-ZrO_2_), which stabilizes lattice oxygen chemistry. Fang et al. showed that porous t-ZrO_2_ promotes oxygen spillover to IrO_x_ nanoclusters, enabling faster OER kinetics and improved structural stability (Figure 2G). (Fang et al., 2025) The resulting PEM electrolyzer operated stably for 1,600 h at 1 A cm-2 with an ultralow Ir loading of 0.1 mgIr cm-2. CeO_2_-based electron-buffer supports illustrate a different mechanism. In the Ir-Ce substitutional solid solution, electron donation from the matrix stabilized lower-valence Ir and suppressed oxidative dissolution (Figure 2H). (Dong et al., 2024) Compared with ZrO_2_-mediated oxygen spillover, this strategy regulates Ir redox state more directly. Emerging oxides should therefore be judged by whether lattice confinement, oxygen transfer, or charge buffering remains effective in a conductive and scalable catalyst layer.

Overall, other oxide-supported Ir catalysts are valuable because they show that the support can be used to engineer the intrinsic chemistry of Ir active sites, not just their spatial distribution. Their main opportunity lies in exploiting support-induced orbital interactions, lattice confinement, and mixed-oxide stabilization. Their central challenge is that gains in one property, such as site stability, often come at the expense of another, such as conductivity, synthetic simplicity, or scalability. As a result, these systems are likely to play a dual role in the field: some may evolve into practical support platforms, whereas others will remain important mechanistic model systems for understanding how support chemistry governs Ir oxidation-state evolution, oxygen intermediate binding, and dissolution pathways under PEMWE conditions.

### 2.4 Design principles emerging from supported Ir catalysts

Across support families, four principles emerge. First, dispersion raises Ir utilization but not necessarily durability. Nanoparticles, subnanoclusters, and isolated atoms expose more Ir than bulk IrO_2_, yet low coordination also facilitates over-oxidation, migration, aggregation, and dissolution (Moriau et al., 2022; Yang et al., 2025; Yuan et al., 2023). The objective is a stable density of working-state sites, not the smallest initial particle size. Second, structure and composition influence OER differently. Continuous IrO_x_ shells and epitaxial IrO_2_ networks mainly improve connectivity and anchoring; lattice-embedded Ir changes Ir-O-Ir geometry and intermediate binding; oxygen-spillover and electron-buffer interfaces modify oxygen exchange and Ir valence (Dong et al., 2024; Fang et al., 2025; Kost et al., 2024; Li et al., 2025). These effects may change the balance between adsorbate-evolution and lattice-oxygen pathways, but pathway assignment requires operando or isotope evidence rather than activity trends alone (Zhang et al., 2025). Third, the support is dynamic: dopant loss, reconstruction, or dissolution can change conductivity and detach stabilized Ir (Jang et al., 2022). Finally, half-cell mass activity is insufficient for practical ranking. At low loading, through-plane conductivity, ionomer coverage, pore connectivity, water supply, and oxygen removal determine catalyst utilization (Xu et al., 2025; Zhang et al., 2022). A credible support must preserve Ir and support integrity while maintaining cell voltage under steady and dynamic operation.

## 3 Conclusions and perspectives

Supported Ir-based catalysts have become a major focus in PEMWE research because they provide a practical route to lowering Ir loading while maintaining activity and durability. As discussed throughout this mini-review, catalyst performance depends not only on the intrinsic properties of Ir, but also on how the support regulates its dispersion, local structure, redox behavior, and stability under acidic OER conditions. Among the available systems, oxide-supported Ir catalysts remain the most promising for PEMWE anodes because oxide supports offer the most practical balance between corrosion resistance and compatibility with strongly acidic anodic environments. In particular, TiO_2_- and SnO_2_-based systems have established the fundamental design framework for supported Ir catalysts. In parallel, recent work on highly dispersed Ir clusters and single-atom sites has improved Ir utilization and advanced understanding of support-controlled activity and stability. These advances make clear that catalyst behavior is increasingly governed by the Ir-support interaction rather than by the intrinsic properties of Ir alone.

Three unresolved gaps define the field. First, conductivity, corrosion resistance, and Ir anchoring rarely improve simultaneously, and initial structural stability does not guarantee a stable working state. Second, reconstruction, lattice-oxygen exchange, support dissolution, and Ir dissolution are often measured separately, obscuring the origin of long-term decay. Third, catalysts are frequently ranked by mass activity in thin model electrodes, whereas low-Ir MEAs are limited by areal loading, catalyst-layer resistance, ionomer distribution, porosity, and interfacial transport. Future studies should report cell voltage at relevant current density together with Ir loading, support and Ir dissolution, and durability under steady and fluctuating operation. Only catalysts retaining these metrics simultaneously should be regarded as practical advances.

Overall, supported Ir-based catalysts are not only a strategy for reducing noble-metal loading, but also a broader platform for integrating active-site regulation, support chemistry, interfacial control, and electrode design. Continued progress will depend on linking atomic-scale understanding with realistic device-level evaluation, ultimately enabling durable low-iridium PEM water electrolyzers for large-scale green hydrogen production.
